# Advances and Challenges in mRNA Cancer Vaccines: Individualized Design and Delivery

**DOI:** 10.3390/bioengineering13101153

**Published:** 2026-10-02

**Authors:** Liyun Huang, Yuting Tang, Xiaobing Chen, Zhen Wu, Junlan Xiao, Qi Xiang, Huajian Tian

**Affiliations:** 1 Department of Basic Medicine, Gannan Health Vocational College, Ganzhou 341000, China; aniony@163.com (L.H.); tangyuting@gnhvc.edu.cn (Y.T.); baiihrgw11@163.com (X.C.); 2 Ganzhou Key Laboratory of Targeted Therapy and Molecular Pathology, Ganzhou 341000, China; wuzhen@mail.gzsrmyy.com (Z.W.); xiaojunlan@mail.gzsrmyy.com (J.X.); 3 Ganzhou Hospital-Nanfang Hospital, Southern Medical University ( Ganzhou People’s Hospital), Ganzhou 341000, China; 4 Guangdong Provincial Key Laboratory of Bioengineering Medicine, Institute of Biomedicine, Jinan University, Guangzhou 510632, China; 5 Biopharmaceutical R&D Center of Jinan University Co., Ltd., Guangzhou 510632, China

**Keywords:** mRNA cancer vaccines, neoantigens, structural engineering, machine learning (ML), lipid nanoparticles (LNPs)

## Abstract

Since the widespread use of mRNA-based COVID-19 vaccines, significant progress has also been made in mRNA cancer vaccines. Currently, mRNA cancer vaccines represent one of the most promising treatments for advanced tumors, with several candidates in clinical trials. Compared with traditional treatment methods, mRNA cancer vaccines have several advantages. However, several challenges of mRNA cancer vaccines remain, including the accurate and rapid identification of neoantigens and hotspot mutations that match different HLA types. To address these challenges, we review four key aspects of mRNA cancer vaccine development. These include neoantigen identification technologies, machine learning and AI prediction, mRNA sequence and structure design, and the development of LNP delivery systems. Additionally, we analyze the pipelines of leading companies in this field, providing insights into personalized mRNA cancer vaccines. This review also discusses future challenges and prospects, aiming to help guide this promising field toward the full realization of its potential.

## 1. Introduction

Cancer remains the foremost health challenge worldwide and is also one of the main causes of death. Over the past few decades, the field of oncology has undergone profound changes, particularly with the rapid elucidation of tumor immune mechanisms and the concurrent progress of cellular immunotherapy [1,2]. In this situation, cancer vaccines have become a crucial treatment. The research on cancer vaccines spans over 20 years, evolving through three primary technological iterations: peptide-based vaccines [3], dendritic cell (DC) vaccines [4], and mRNA vaccines [5].

Although mRNA vaccines have been under investigation for decades for infectious diseases and oncology, the SARS-CoV-2 pandemic served as the ultimate catalyst for their large-scale validation [6,7]. The urgency of the COVID-19 outbreak initiated research focused on rapid deployment of high-efficacy, mass-producible vaccines. By December 2020, the FDA granted Emergency Use Authorization to three vaccines, two of which utilized mRNA encoding the viral spike protein encapsulated in lipid nanoparticles to facilitate systemic delivery [8].

The modern success of mRNA technology is fundamentally attributed to the research work of 2023 Nobel laureates Drew Weissman and Katalin Karikó. They have standardized this field through nucleotide modification; specifically, replacing uridine with 1-methylpseudouridine [9] and the implementation of lipid nanoparticle (LNP) delivery systems revolutionized the field [10].

In this review, we present a novel perspective to elucidate the current characteristics and challenges of mRNA cancer vaccines. First, we benchmark leading AI/ML neoantigen prediction platforms against key metrics: predictive accuracy, HLA allele coverage, and inherent limitations. Second, we critically appraise mRNA engineering strategies, including codon optimization, UTR design, capping chemistry, and platforms such as linear mRNA, saRNA, and circRNA. Third, we analyze LNP formulation principles and their mechanistic links to biodistribution, cellular uptake, endosomal escape, and targeted delivery. Last, we evaluate pivotal clinical trial data and synthesize translational barriers spanning neoantigen discovery, manufacturing scalability, and regulatory pathways.

## 2. Advantages of mRNA Cancer Vaccine

**Figure 1:**
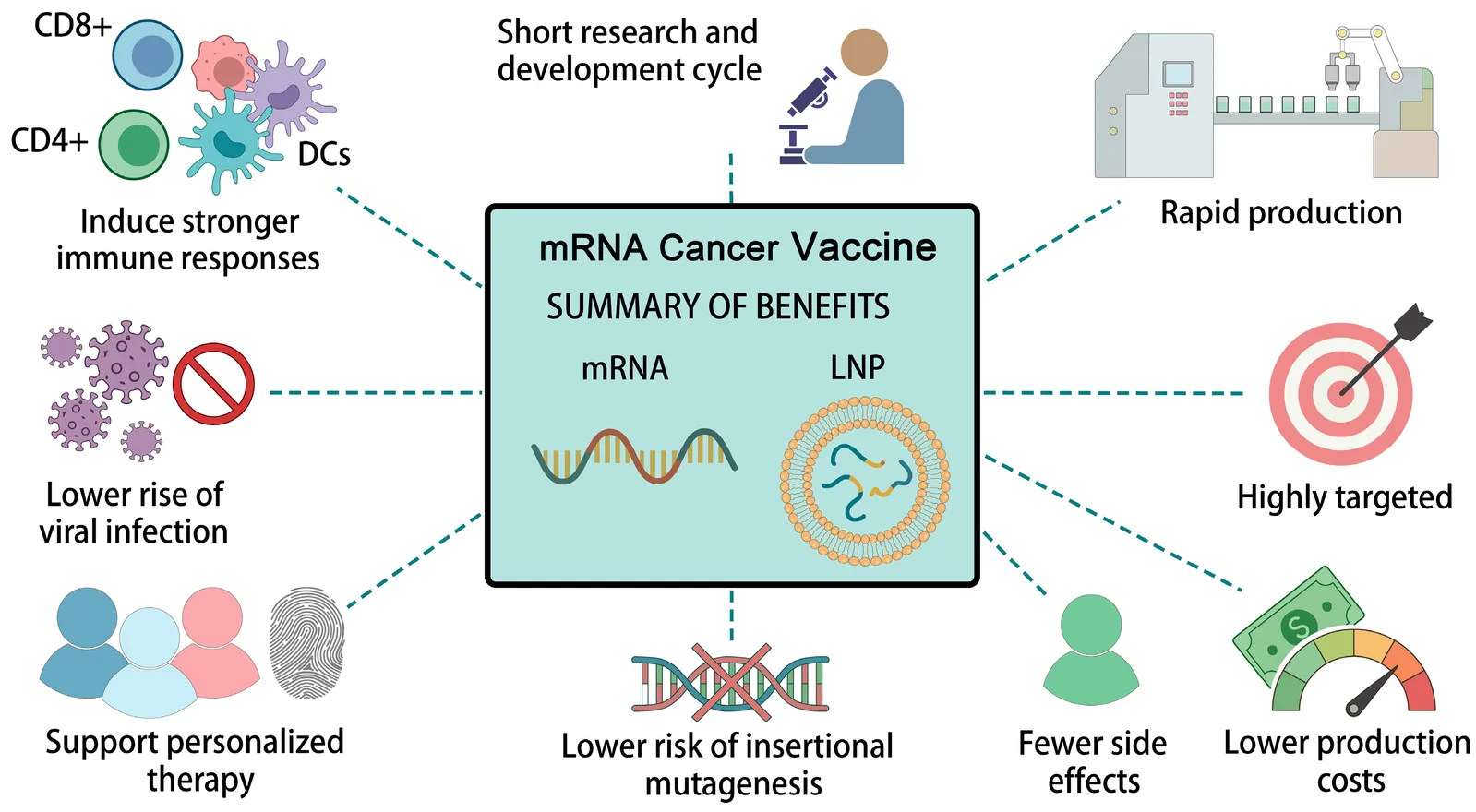
The key advantages of mRNA cancer vaccines. Advantages of mRNA cancer vaccines include a short research and development cycle, rapid and lower production costs, high specificity, effective immune system activation, minimal side effects, and no risk of genomic integration or viral infection, making them an optimal choice for personalized therapy. (Figure was drawn in Adobe Illustrator v29.x).

mRNA cancer vaccine offers a novel, rapid, precise, and flexible approach for cancer immunotherapy. Figure 1 summarizes the main advantages of mRNA cancer vaccines. The most notable feature is the rapid progress during the research, development, and production processes. As long as the sequence of the neoantigens is known, personalized vaccines can be customized and manufactured within a few weeks in accordance with Good Manufacturing Practice (GMP) guidelines. This is faster than traditional peptide vaccines [11,12]. This flexibility helps to achieve high personalization—tailoring the vaccine design based on the specific mutation patterns of each tumor, thus truly realizing precision medicine [13].

In terms of immunogenicity, after mRNA vaccine injection, they can simultaneously activate CD8+ T cells (cytotoxic immunity) and CD4+ T cells (helper immunity), and promote B cells to produce antibodies, thereby inducing a strong and broad-spectrum immune response [14]. In terms of safety, mRNA does not enter the cell nucleus, thus avoiding potential insertional mutagenesis or oncogenesis, which is safer than DNA vaccines [15].

Furthermore, since mRNA cancer vaccines have controllable immunogenicity and no neutralizing antibodies against the delivery vector, they are suitable for repeated administration, which facilitates multiple booster vaccinations. They can also be easily combined with other treatment methods, such as immune checkpoint inhibitors or antibodies [16]. Benefiting from their versatility and scalability, mRNA vaccine technology has been widely applied in cancer immunotherapy, with multiple platforms entering clinical development.

In summary, mRNA cancer vaccines offer certain advantages compared with peptide-based or dendritic cell vaccines. Design and production take only 4–6 weeks, compared to 3–6 months for DC vaccines. They may encode many neoantigens at once, thereby enabling wide immune coverage without genomic integration. LNP-mediated innate immune activation has its own adjuvant effect. Manufacturing based on in vitro transcription is economically feasible and readily scalable [5,17,18]. Such advantages strongly support the idea of mRNA as a leading platform for future personalized cancer immunotherapy. Nonetheless, several major issues related to neoantigen selection, mRNA quality, and delivery still need to be solved if this potential is to become reality.

## 3. Neoantigen Identification

### 3.1. Classification of Tumor Antigens

Tumor-associated antigens (TAAs) are self-antigens that are highly expressed in tumor cells but rarely in normal tissues. Representative TAAs include HER2, gp100, WT1, NY-ESO-1, and MAGE-A3 [19,20]. Since TAAs are self-proteins, they may provoke central immune tolerance or cause autoimmune-related complications. TAAs are the main candidates for off-the-shelf (non-personalized) anti-cancer vaccines, as their antigenic identity is common to many individuals with the same type of cancer.

Tumor-specific antigens (TSAs) exhibit much higher tumor specificity, as they are derived from genetic mutations (e.g., mutant KRAS or p53) [21], viral integration (e.g., HPV E6/E7) [22], or aberrant splicing events [23]. They are expressed exclusively in tumor cells and are completely absent from normal tissues. TSAs can more effectively activate CD8+ cytotoxic T lymphocytes, elicit robust anti-tumor immune responses, and bypass central immune tolerance. Most TSAs are also referred to as neoantigens, and personalized cancer vaccines are primarily developed around these neoantigens [24].

At present, multiple industry-leading companies have mRNA cancer vaccines in clinical trials, featuring pipelines directed against both TAA and TSA, and are being developed for a range of cancer indications (Table 1).

### 3.2. Recent Technological Approaches for Neoantigen Identification

The rapid development of high-throughput sequencing and bioinformatics has largely transformed neoantigen discovery. The majority of cancer vaccine research mainly relies on next-generation sequencing (NGS) [31]. By conducting sequencing in both normal and tumor tissues of patients, researchers are able to identify somatic mutations and use predictive algorithms to prioritize the selection of candidate neoantigens [32]. A typical example is Autogene cevumeran, an individualized mRNA vaccine developed by BioNTech for refractory pancreatic ductal adenocarcinoma [16]. This personalized vaccine is engineered to encode up to 20 patient-specific neoantigens, tailored to the unique mutational profile of each individual [33].

Although NGS has significant advantages, it is increasingly being complemented by immune-peptidomics in the detection of surface-displayed antigens [34,35]. This method is based on mass spectrometry (MS). By enriching peptide–MHC (pMHC) complexes through immunoprecipitation from tumor lysates, the peptide segments bound to MHC can be detected by LC-MS/MS [36]. In order to enhance the accuracy of neoantigen recognition, mass spectrometry data is usually combined with genomic information. In this collaborative workflow, the mutation libraries constructed by NGS can serve as a customized reference for MS spectral search [37]. The typical approach is to construct a patient-specific mutant peptide library through sequencing, then use this library to interpret mass spectrometry spectra. This method can obtain more accurate information on neoantigens [38,39]. Another approach employs deep learning technology to study a large dataset of HLA peptides and build a genomic characteristics model, constructing a peptide presentation computational model for predicting neoantigens [40].

Despite this progress, several practical problems remain. Due to the heterogeneity of cancer, neoantigens identified from a single biopsy may not represent the whole tumor antigen landscape, which may lead to incomplete immune coverage [41,42]. Prediction itself is the next weak point. A peptide that binds HLA with high affinity is not automatically a therapeutically useful neoantigen. Whether it provokes a T-cell response depends on the patient’s TCR repertoire, on processing efficiency, and on antigen stability [43,44]. HLA diversity poses another significant challenge. More than 12,000 HLA class I alleles have been described, and current algorithms work well only for the best-studied ones such as HLA-A*02:01. For rarer alleles, particularly those frequent in non-Caucasian populations, coverage remains poor [42,45]. False positives are also common. Candidate neoepitopes picked out by computational pipelines often fail experimental validation. Each failure consumes manufacturing capacity on a vaccine that may never work [46]. Validation is the final bottleneck. T-cell assays, tetramer staining, and intracellular cytokine staining are slow and labor-intensive, and they sit on the critical path of every personalized vaccine workflow [43].

### 3.3. Application of Machine Learning and AI Algorithms

Regardless of whether peptide information is obtained through sequencing or mass spectrometry analysis, using artificial intelligence models to predict neoantigen peptides that match specific HLA subtypes is essential. Various algorithms have been proposed to score and rank neoantigens based on their likelihood of presentation on a patient’s HLA molecules and their potential recognition by high-affinity, T-cell clone-specific responses [47,48].

With the emergence of thousands of datasets of neoantigen immunogenicity measurements, machine learning (ML) methods can be utilized to train robust immunogenicity prediction algorithms. Several neoantigen prediction algorithms based on machine learning have been developed and widely adopted. A comprehensive comparison of the major platforms is presented below:

NetMHCpan-4.0 is one of the most frequently applied prediction tools, using both eluted ligand (EL) and peptide-binding affinity (BA) data to forecast peptide–MHC class I interactions [47]. It has been trained on 85,217 measurements of peptide–MHC binding affinity, so it treats all HLA-A, HLA-B, and HLA-C alleles with known sequences as covered. NetMHCpan-4.0 gives an area under the ROC curve (AUC) in the range of 0.85–0.90 for HLA-A*02:01 and for many well-characterized alleles; performance is worse for rarer alleles because of small training sets. A major limitation is its use of binding affinity as a stand-in for immunogenicity-which ignores later-stage T-cell recognition.

MHCflurry 2.0 improves upon the earlier version by including an assessment of antigen processing, thus making pan-allele prediction of MHC class I ligands feasible. Trained on more than 240,000 individual mass spectrometry-derived ligand data points, it now unifies proteasomal cleavage, TAP transport, and MHC binding within one predictive model. It gives results comparable to NetMHCpan-4.0 on standard benchmarks but achieves quicker inference thanks to its neural network design [49]. However, in line with NetMHCpan, it shows allele-specific performance-reduced accuracy for underrepresented HLA alleles or non-Caucasian backgrounds.

DeepHLA uses deep learning-specifically convolutional neural networks-to predict HLA-peptide binding and to extract features based on amino acid sequences [48]. Although it gives better results than position-specific scoring matrix(PSSM)-based models, its training dataset is not as large as that of NetMHCpan or MHCflurry, which limits its utility for new HLA alleles.

AlphaFold-assisted prediction of structure has now become a complementary method, using the structural power of AlphaFold to estimate peptide–MHC configurations and to deduce binding from such compatibility [50]. Such an approach yields atomic-level information about peptide–MHC interactions but is costly to implement and lacks large-scale clinical validation for neoantigen use.

Transformer-based models represent the latest advancement in neoantigen prediction. In 2024, a research team led by Li et al. reported a neural network algorithm for predicting TCR–antigen–HLA binding and screening of neoantigens [51]. By developing the Sliding Transformer (PISTE) algorithm, this study achieved accurate prediction of the TCR–antigen–HLA interaction, which effectively supported the screening of neoantigens. This algorithm introduces a novel physical design into the model, simulating a virtual process of two or more biological sequences in combination. This provides a new perspective and methodology for exploring the complex TCR/antigen/HLA interactions and the molecular basis of human immune responses. PISTE was tested on several immunogenic tumor types and successfully identified neoantigens. In a prospective study of prostate cancer, six out of eight patients (75%) mounted T-cell responses against the PISTE-predicted neoantigens, supporting its clinical feasibility.

Building models by machine learning and developing AI algorithms is particularly critical for the development of mRNA cancer vaccines. Currently, leading global companies, including BioNTech and Moderna, have established proprietary data models and algorithms to screen and evaluate sequenced mutant genes, predicting the possibility of serving as neoantigens [33,52].

The identification and prioritization of neoantigens through the AI-assisted approaches described above directly informs the customized design of mRNA sequences encoding these targets, which is the focus of the next section.

## 4. mRNA Sequence and Structural Optimization

### 4.1. Design of UTRs

**Figure 2:**
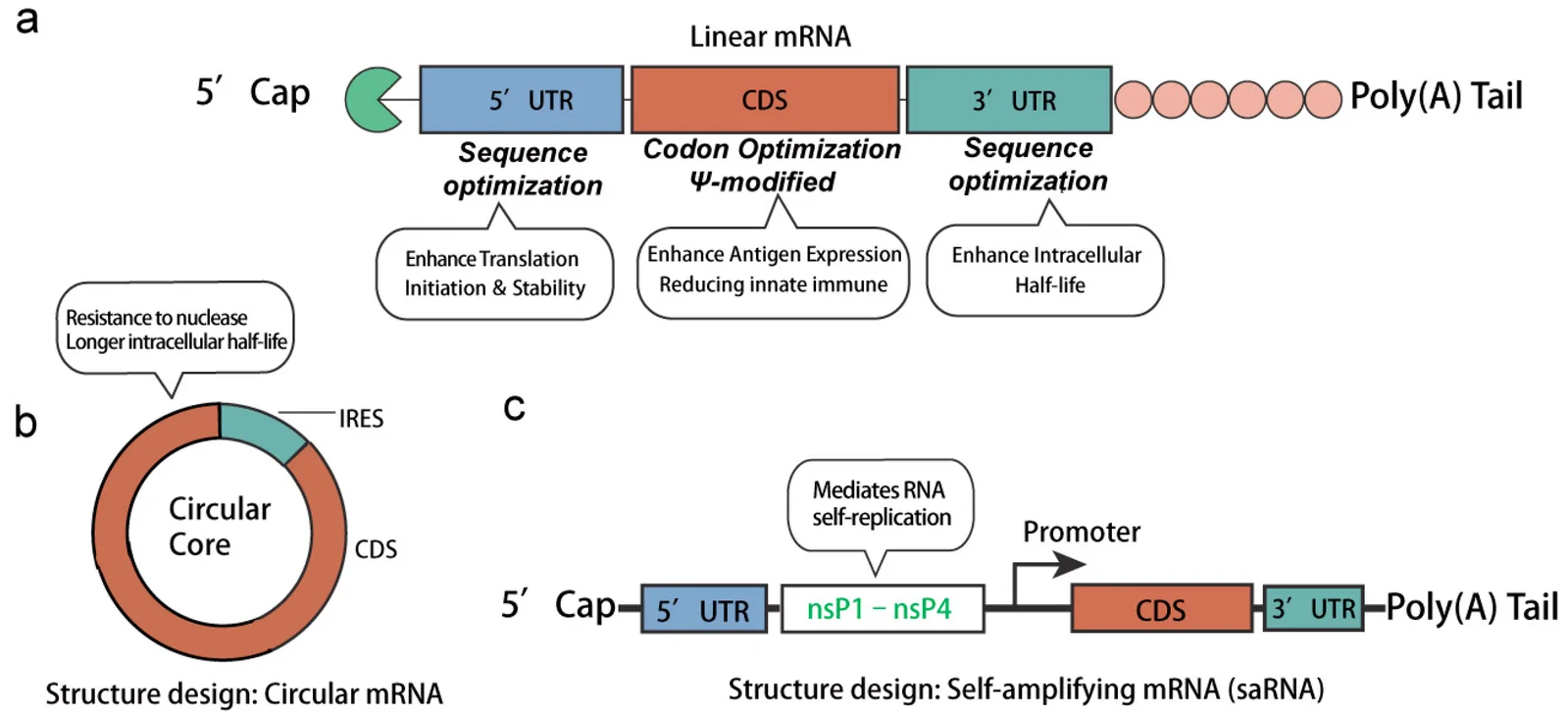
Schematic illustration of mRNA sequence optimization and structural design. (**a**) Several optimization strategies are employed to improve its performance: 5′ UTR optimization enhances translation initiation and stability; codon optimization of the CDS increases antigen expression efficiency; pseudouridine incorporation reduces innate immune recognition and improves intracellular half-life; 3′ UTR optimization increases mRNA stability, prolongs intracellular half-life, and enhances resistance to nuclease degradation. (**b**) The circular structure confers enhanced stability and resistance to exonuclease-mediated degradation, leading to a longer intracellular half-life and sustained protein expression compared to linear mRNA. (**c**) nsP1–nsP4 are derived from the alphaviral replicase and are placed upstream of the CDS. These nsP genes encode a replicase complex that mediates RNA self-replication within the host cell, resulting in massive amplification of the CDS transcripts. (Figure was drawn in Adobe Illustrator v29.x).

mRNA cancer vaccine development is closely linked to the structural design of mRNA-particularly with regard to stability, translation efficiency, and immunogenicity. Optimizing mRNA involves attention to a number of basic features, namely the 5′ cap structure, 5′ and 3′ untranslated regions (UTRs), open reading frame (ORF), and poly(A) tail [52]. Of these features, the efficiency of 5′ capping has a definite influence on mRNA stability and on translation initiation. Optimization of the UTR sequence may help control both the half-life and the rate of translation [53] (Figure 2a).

Current strategies for UTR engineering can generally be classified into two categories. The first strategy involves the selection of naturally occurring UTR sequences derived from mammalian or viral sources, which are typically obtained from highly expressed genes. Commonly used mammalian UTRs are derived from human alpha-globin (HBA) [54], human beta-globin (HBB) [55], and human heat shock protein 70 (HSP70) genes [56]. Additional mammalian sources include murine alpha-globin genes [57]. Viral UTRs are frequently derived from Venezuelan equine encephalitis virus (VEEV) [58] or Sindbis virus (SINV) [59].

The second strategy is one of de novo designs—using either computational or deep learning methods—for UTR sequences. Models were built upon polysome profiling of fully or partially randomized 5′ UTR collections and applied to help find good 5′ UTRs by means of gradient descent or of generative neural networks [60]. Such data-oriented techniques now seem capable of surpassing natural UTRs in many respects, though their use in medicine awaits more thorough testing and validation.

### 4.2. Codon Optimization

Codon optimization is one of the key determinants of translation efficiency for mRNA vaccines. Codon choice has to meet multiple conditions. Chosen codons need to correspond with the normal frequency of host tRNA usage. The percentage of GC content should not be too great (ideally 30–70%), nor should long runs of identical nucleotides occur. Structural elements near the start of translation must be kept stable, since otherwise ribosome scanning is impeded [54]. Too much GC content may paradoxically hinder translation by forming stable RNA secondary structures, while too little GC content may lead to mRNA instability. Computational tools like mRNA designer now exist for the integrated optimization of such parameters. Additionally, the codon-optimization approach has to avoid cryptic splice sites, premature poly(A) signals, and immunologically active sequence patterns (that might cause innate activation).

### 4.3. Nucleotide Modification

In addition to optimization of RNA structural elements, the RNA molecule itself also requires further engineering. The application of unmodified mRNA in mRNA-based vaccines is limited by several intrinsic drawbacks, including its susceptibility to extracellular ribonuclease (RNase)-mediated degradation and its inherent immunogenicity [54,61]. Katalin Karikó and colleagues reported that incorporation of pseudouridine (Ψ) into mRNA significantly enhances the translational efficiency of the encoded protein compared with unmodified mRNA [9]. In addition, Ψ-modified mRNA does not induce the production of pro-inflammatory cytokines, such as IFN-α and TNF-α, indicating markedly reduced immunogenicity relative to unmodified mRNA. Pseudouridine modification is thought to prevent recognition of mRNA by Toll-like receptors (TLRs) and protein kinase R (PKR), thereby reducing innate immune activation while improving translational efficiency [62].

In addition to pseudouridine (Ψ), other modifications can be introduced through the methylation of RNA at different nucleotide sites, including 5-methylcytosine (m5C), N1-methyladenosine (m1A), N6-methyladenosine (m6A), 1-methylguanosine (m1G), 2-methylguanosine (m2G), and 7-methylguanosine (m7G) [63]. Among them, m5C has attracted much attention due to its ability to enhance translation efficiency and reduce immunogenicity [64].

### 4.4. 5′ Cap Chemistry and Capping Efficiency

Every functional mRNA carries a 5′ cap. This small modification governs stability, splicing, nuclear export, and translation initiation, the last through its contact with eukaryotic initiation factor 4E (eIF4E). In vitro transcription (IVT) mRNA can be capped in one of two ways. The enzymatic route uses vaccinia virus capping enzyme together with 2′-O-methyltransferase, and yields the natural Cap 1 structure (m7GpppN1m). This cap is identical to the one that was barely noticed by innate immune sensors. The price is extra enzymatic reactions and extra purification. The alternative is co-transcriptional capping with CleanCap reagents [65]: the cap is added as transcription proceeds, in a single step, which simplifies manufacturing considerably. How well the reaction works matters a great deal. Uncapped mRNA is quickly destroyed by cellular 5′ exonucleases, and what survives can trip innate immune sensors such as RIG-I. Reported efficiencies fall between 70 and 95%, with the enzymatic route usually above 90% and co-transcriptional methods somewhat lower. Both LC-MS/MS and ribozyme-based assays can measure capping efficiency directly [66].

### 4.5. Poly(A) Tail Design

The poly(A) tail at the 3′ end of an mRNA has major roles; among them are stability of the molecule, translation efficiency, and transport across nuclear membranes. The ideal length of the poly(A) tail for IVT mRNA vaccines is generally 100–150 nucleotides, a value that balances stability with translational benefit [52]. Two general approaches are available for incorporation of the poly(A) tail. The first utilizes direct encoding of the poly(A) sequence in the DNA template, thus guaranteeing length control, but plasmid instability during growth in bacteria may be limiting. The second uses enzymatic polyadenylation, which permits variable tail lengths but may give heterogeneous results. The poly(A) tail binds to poly(A)-binding protein, which joins eIF4G/eIF4E to the 5′ cap in a loop-like fashion, thereby helping ribosomes recycle and translate efficiently. Additionally, the poly(A) tail prevents or delays deadenylation-dependent decay of mRNA, and its length correlates closely with mRNA half-life in transfected cells [54].

### 4.6. dsRNA Impurities, Purification, and Innate Immune Sensing

In vitro transcription has an unwelcome side effect: the reaction spits out double-stranded RNA (dsRNA) byproducts alongside the intended product. Cells read those impurities as a virus. RIG-I, MDA5, TLR3, and PKR all fire, type I interferon follows, and both translation and the mRNA itself take the hit [67]. Protein yields sink. So dsRNA removal is not optional in manufacturing; it decides how much protein a dose can produce.

Cellulose-based chromatography handles the problem cheaply. dsRNA sticks to cellulose, single-stranded mRNA flows through, and the two separate. Reverse-phase high-performance liquid chromatography (RP-HPLC) resolves dsRNA more finely at higher cost [68]. A third tactic attacks the impurity before it forms: hydrophobic interaction chromatography cleans dsRNA off linearized plasmid DNA templates before IVT runs, cutting dsRNA formation at the source. Similar contaminant concerns apply to synthetic circRNA, where recent purification work aims to bring impurity levels low enough to avoid cellular dysfunction [69].

### 4.7. Structure Design: Comparative Analysis of saRNA, circRNA, and Linear mRNA

Rapid degradation is a major obstacle to long mRNA half-life, and so limits immune activation [70]. To help achieve both stability and effectiveness for mRNA-based cancer vaccines, a range of structural modifications has been proposed. Circular RNA (circRNA) is now regarded as an interesting form of mRNA, since it lacks a cap, is less vulnerable to nuclease attack, and persists longer within cells, thus having a longer intracellular half-life [67,71] (Figure 2b). Such features seem likely to facilitate prolonged antigen exposure and powerful T-cell responses [72].

Self-amplifying RNA (saRNA) shares a basic structural organization similar to that of conventional linear mRNA; however, it is capable of intracellular self-replication through a replicase located downstream of the 5′ untranslated region (5′UTR) [15]. saRNA vectors are typically derived from alphaviruses, in which viral pathogenic genes are removed while the nonstructural protein-coding sequences (nsP1–nsP4) are retained upstream of the target gene [73] (Figure 2c). These nonstructural proteins collectively form an RNA-dependent RNA polymerase complex that mediates RNA replication. This mechanism reduces the required saRNA dose in cancer vaccine production [74].

It is important to note that conventional mRNA, saRNA, and circRNA platforms differ substantially in their preclinical and clinical maturity. Conventional mRNA is the most clinically advanced platform, validated by the global deployment of COVID-19 vaccines and currently being tested in multiple cancer vaccine clinical trials (e.g., mRNA-4157, BNT111, BNT122). saRNA has demonstrated promising results in preclinical cancer models and has entered early-phase clinical trials, including the ChAd68–samRNA neoantigen vaccine [75]. However, saRNA’s larger payload size and potential for sustained innate immune activation through replication intermediates present unique challenges for clinical translation. circRNA vaccines remain primarily in the preclinical stage for cancer applications [67,72], with ongoing optimization of cyclization efficiency, purity, and scalability. Table 2 provides a detailed comparison of these three platforms across key parameters including stability, translation efficiency, payload capacity, manufacturing complexity, immune activation profile, and clinical maturity. The choice of platform should be guided by the specific application: conventional mRNA for rapid, well-validated responses; saRNA for dose-sparing and sustained expression; and circRNA for applications requiring prolonged antigen presentation.

The optimization of mRNA sequence and structure, as discussed in this section, provides the molecular blueprint for effective cancer vaccines. However, the translation of these optimized mRNA molecules into clinical efficacy depends critically on their delivery to target cells, which is the subject of the next section on lipid nanoparticles.

## 5. Lipid Nanoparticles

### 5.1. Application of Lipid Nanoparticles

The success of mRNA vaccines largely depends on the development of lipid nanoparticle technology. Before LNP-based delivery systems, cancer vaccines were primarily limited to peptide vaccines or dendritic cell (DC) vaccines [69,76]. LNP is regarded as the most advanced delivery platform for mRNA vaccines because it can protect the mRNA from enzymatic degradation and deliver mRNA into cells [77]. Whether the results obtained in preclinical studies or clinical trials, they have proved that LNP was a key component in the development of effective mRNA cancer vaccines [18].

**Figure 3:**
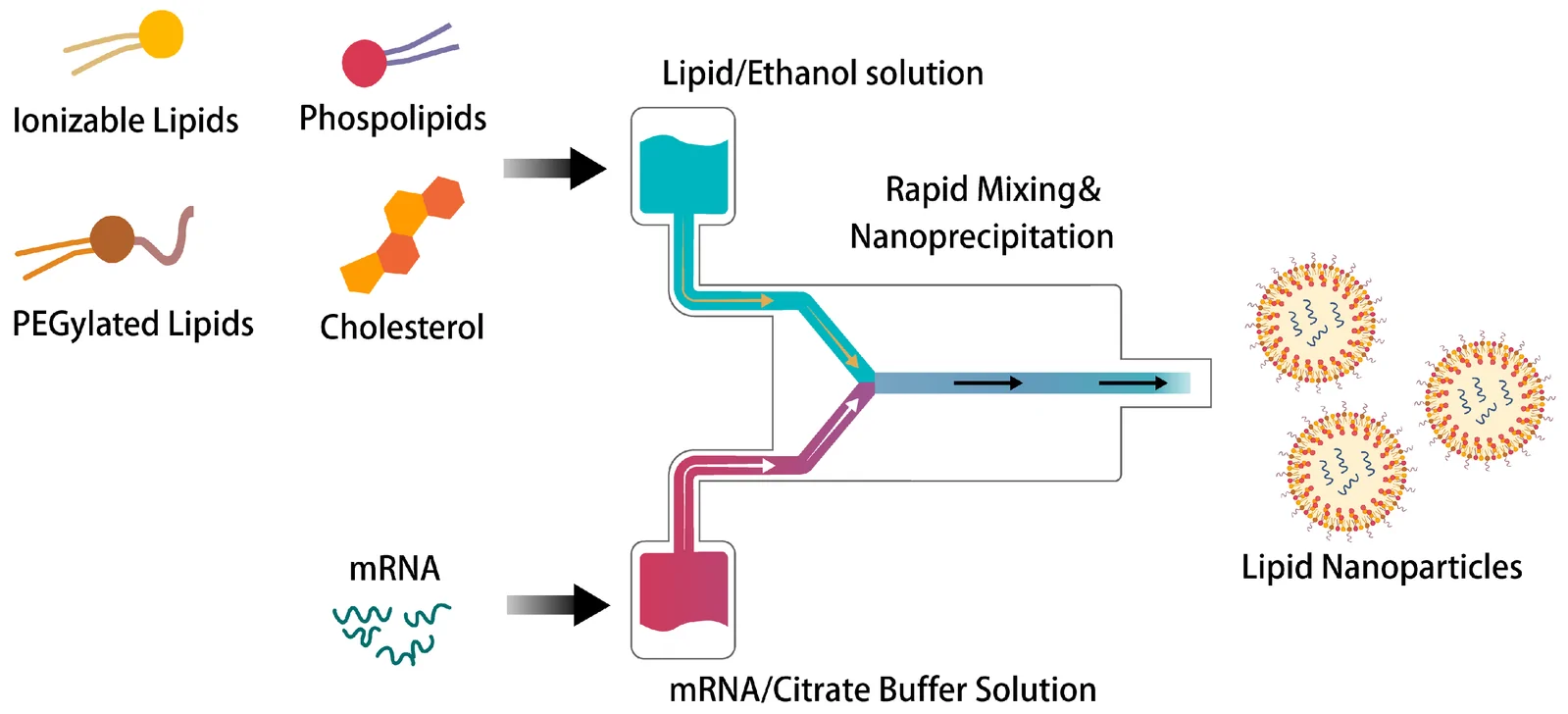
Schematic diagram of the LNP manufacturing process. Lipid components (ionizable lipids, phospholipids, cholesterol, and PEGylated lipids) are dissolved in ethanol, while mRNA is dissolved in an acidic aqueous buffer. The two solutions are rapidly mixed via microfluidic mixing, inducing nanoprecipitation and self-assembly into uniform lipid nanoparticles (~100 nm) that encapsulate mRNA within the core. (Figure was drawn in Adobe Illustrator v29.x).

Lipid nanoparticles (LNPs) were originally developed as delivery vehicles for small interfering RNA (siRNA) therapeutic drugs [78]. In August 2018, the patisiran developed by Alnylam was granted its first approval by the US Food and Drug Administration (FDA). LNPs have been proven to have significant efficacy in mRNA delivery and have played an indispensable role in the rapid development of COVID-19 vaccines [79]. Their high transfection efficiency and inherent adjuvant activity can induce a strong immune response. LNPs are nano-sized lipid carriers with a diameter of approximately 100 nm. They are typically composed of ionizable lipids and helper lipids (Figure 3), including phospholipids, cholesterol, and polyethylene glycol (PEG)-modified lipids [80]. The composition and physical properties of these lipids have significant influences on the biological distribution, cellular uptake, endosomal escape, immunogenicity, and delivery efficiency [81]. The rational design and optimization of LNP formulations are critical for the application of mRNA cancer vaccines.

### 5.2. LNP Composition and Design Principles

All the lipid elements of an LNP perform certain functions, and their logical design is very important for vaccine success:

Ionizable lipids are the central functional component, responsible for mRNA packaging, cell entry, and endosomal escape. Their pKa is a key parameter. Values between 6.0 and 6.5 preserve neutrality of the lipids at normal physiological pH, minimizing serum binding and toxicity. In an acidic endosomal setting (pH about 5.5), the lipids acquire a positive charge. This charge change favors membrane rupture and RNA release [81,82]. The now-approved Pfizer-BioNTech COVID-19 vaccine makes use of ALC-0315, whereas the Moderna vaccine employs SM-102, both of which are well adapted to this pH-regulated ionization mode. Recent progress has included the development of biodegradable ionizable lipids with ester or disulfide linkages that degrade inside cells, avoiding long-term accumulation upon multiple uses [83,84]. Such next-generation lipids are aimed at solving the safety issues raised by chronic lipid buildup in the liver or spleen after many doses.

PEG–lipids (including ALC-0159 from the Pfizer vaccine and PEG2000-DMG from the Moderna vaccine) serve as a hydrophilic stealth coating that minimizes both opsonization and nonspecific uptake, thus prolonging circulation time. PEGylation is not without drawbacks. Anti-PEG antibodies may appear with multiple administrations, leading to rapid blood clearance as well as diminished potency [85]. PEG–lipids seem to interfere with uptake by cells or with endosomal escape; there is a trade-off between stability and efficiency. In some cases, PEG-related hypersensitivity has been documented clinically. Alternatives to PEG—such as polysarcosine—now seem promising for overcoming these problems [86].

Cholesterol is involved in membrane fluidity and in its stability—both of which affect the phase transition temperature of the lipid bilayer and endosomal escape efficiency. The molar proportion of cholesterol has a marked effect on LNP morphology and on delivery efficiency [82]. Helper phospholipids help form an intact bilayer and assist in membrane fusion when endosomal escape occurs. The kind of phospholipid head group and the acyl chain length influence the phase transition temperature and fusogenicity of the LNP [87].

Among the factors that influence both the physical/chemical properties and the transfection efficiency of LNPs is the molar ratio of their lipid components. The clinically approved Pfizer-BioNTech COVID-19 vaccine has ALC-0315 (46.3%), DSPC (9.4%), cholesterol (42.7%), and ALC-0159 (1.6%) [81]. The Moderna COVID-19 vaccine employs SM-102, DSPC, cholesterol, and PEG2000-DMG with a proportion (molar) of 50:10:38.5:1.5 [86]. Other lipid mixtures—along with altered ratios—may prove useful for improving mRNA transport or for boosting vaccine-related immunity.

Lipid nanoparticles (LNPs) are amenable to many preparation methods, namely, microfluidic mixing, pipette mixing, or vortex mixing. Microfluidic mixing is now the most commonly used approach. In that method, the lipid and mRNA substances go through rapid mixing in a microfluidic apparatus (usually a microchannel or T-junction mixer). The lipids themselves are dissolved in ethanol; mRNA is dissolved in some suitable aqueous buffer solution. The two mixtures are handled with care regarding flow speed, ratio, and temperature, thus enabling uniform nanoscale particle formation of known size [82].

### 5.3. Endosomal Escape and Intracellular Delivery

One of the most formidable obstacles to LNP-based mRNA delivery is escape from the endosome. Once internalized through endocytosis, LNPs follow the canonical endolysosomal route: they are first delivered to early endosomes and then to late endosomes, where the pH falls steadily from around 7.4 to roughly 5.5. Under this acidic condition, the ionizable lipids become protonated, which allows them to bind electrostatically to anionic phospholipids embedded in the endosomal membrane. Such binding disturbs the membrane’s bilayer organization and favors non-bilayer arrangements—most notably the inverted hexagonal phase—eventually tearing the membrane apart and releasing the mRNA into the cytosol [87].

The escape partly explains why overall transfection efficiency remains modest even when uptake is efficient. Two recent strategies address this problem directly. One exploits osmotic pressure: the engineered particles take advantage of the natural osmotic swelling of endosomes, so that mechanical stress ruptures the membrane from within. The other introduces movable lipids, whose conformational changes generate enough mechanical force to break through the endosomal barrier.

LNP biodistribution is an essential factor when evaluating cancer vaccine efficacy. After intravenous or intramuscular administration, LNPs reach multiple organs but tend to accumulate in the liver due to ApoE-related interactions and hepatocyte uptake. For cancer vaccines, it is very advantageous to direct LNPs toward the lymph nodes (sites of antigen-presenting cells: APCs). Selective Organ Targeting (SORT) has led to new strategies for organ-specific LNP delivery using charged modifiers: anionic SORT lipids favor the spleen (an immune-cell-rich organ), while cationic ones may be directed to the lungs [88]. Recent developments of retinoic-acid-based ionizable lipids now make spleen-targeted antigen expression feasible, improving vaccination results [89]. Also, trace PEG–lipid engineering has been examined as a way to obtain albumin-assisted liver targeting with better endosomal escape.

### 5.4. LNP Surface Modifications for Targeted Delivery

Achieving targeted delivery to specific cell types, particularly APCs in lymphoid tissues, is a major goal for cancer vaccine LNP design. Several strategies have been developed: (1) antibody-conjugated LNPs: surface functionalization with antibodies against APC-specific markers (e.g., anti-DEC205, anti-CD40) enables receptor-mediated targeting to dendritic cells; (2) ligand-modified LNPs: incorporation of mannose or CD206-targeting ligands for macrophage/DC targeting [90]; (3) modular assembly approaches: recent advances in modular LNP assembly allow for the facile conjugation of targeting ligands through click chemistry or maleimide–thiol coupling [91]; (4) TLR agonist-conjugated lipids: covalent conjugation of TLR9 agonists to phospholipids has been shown to enhance mRNA-LNP delivery efficiency and dendritic cell cross-priming [92]; (5) silk-based protein corona: a novel approach using silk-based protein coating has demonstrated enhanced mRNA-LNP vaccine efficacy and prevention of tumor relapse [93]. These surface modification strategies represent a rapidly evolving area of research with significant potential to improve the specificity and efficacy of mRNA cancer vaccines.

### 5.5. Safety Considerations and Toxicities

While LNPs have demonstrated an acceptable safety profile in the context of COVID-19 vaccination, several safety considerations are particularly relevant for cancer vaccines, which may require repeated administration: (1) PEG-related immunogenicity: anti-PEG antibodies can develop upon repeated dosing, leading to accelerated blood clearance and potential hypersensitivity reactions [85,94]; (2) innate immune activation: ionizable lipids can induce inflammatory cytokines (IL-6, IL-1β) through various pathways, including inflammasome activation [19], which may be beneficial for vaccine adjuvanticity but could lead to dose-limiting toxicities at higher doses; (3) biodistribution to non-target organs: hepatic accumulation of LNPs and their lipid components may cause liver enzyme elevations, particularly with repeated dosing; (4) biodegradability: non-biodegradable ionizable lipids can persist in tissues for weeks to months, necessitating the development of biodegradable alternatives [83]; (5) manufacturing-related impurities: residual solvents, free lipids, and empty particles must be rigorously controlled through quality assurance processes.

### 5.6. Immunoadjuvant Effects of LNPs

Ionizable cationic lipids are the key factor for the immunostimulatory activities of LNPs. Empty liposomes, prepared with specific ionizable cationic lipids, are administered with influenza protein subunit vaccines into mice. Empty liposomes can act as a potent adjuvant, triggering a strong humoral immune response [10]. In contrast, the empty liposomes prepared using non-ionized cationic lipids failed to exhibit similar adjuvant activity, indicating that not all cationic lipids used for nucleic acid delivery have intrinsic immunostimulatory capacity.

Mechanistically, empty lipid nanoparticles (LNPs) containing ionizable lipids can induce the production of interleukin-6 (IL-6) in the draining lymph nodes and promote the activation of follicular helper T cells (Tfh), enhancing humoral immunity [19]. Furthermore, the adjuvant activity of these LNP formulations was shown to outperform AddaVax, an MF59-like adjuvant that is widely used in vaccine formulations [10].

The development of LNP delivery technologies has created favorable conditions for the clinical use of mRNA cancer vaccines. The next paragraphs will consider, separately or together, the role of such advances in off-the-shelf and in tailored vaccines.

## 6. Development of “Off-the-Shelf” Cancer Vaccine

Tumor-associated antigens (TAAs) are highly expressed in tumor cells but exhibit low or negligible expression in normal tissues, making them the earliest class of targets investigated for universal cancer vaccines. Representative TAAs include HER2 and gp100 [20], WT1, NY-ESO-1, and MAGE-A3 [95]. In addition, several recurrent hotspot mutations, such as KRAS G12V and TP53 R175H, are also considered as shared antigens and may serve as promising targets for off-the-shelf cancer vaccine development [25,96].

The principal advantage of TAAs and hotspot mutations lies in their “shared” property: the same antigen can be expressed across multiple patients with the same tumor type, thereby enabling vaccine manufacturing in advance without the need for individualized customization. BNT116 is an mRNA-based cancer vaccine of BioNTech’s FixVac platform, developed for the treatment of non-small cell lung cancer (NSCLC), particularly advanced or metastatic NSCLC. BNT116 encodes multiple shared tumor-associated antigens commonly overexpressed in NSCLC, including MAGE-A3, CLDN6, KK-LC-1, PRAME, MAGE-A4, MAGE-C1.

The most mature clinical proof of concept for shared-TAA mRNA vaccination comes from BNT111, an intravenously administered RNA–lipoplex (RNA-LPX) vaccine encoding four melanoma-associated shared antigens (NY-ESO-1, MAGE-A3, tyrosinase, and TPTE). In the first-in-human Lipo-MERIT phase I trial (NCT02410733), BNT111 alone or in combination with checkpoint inhibition mediated durable objective responses in patients with checkpoint-inhibitor-experienced unresectable melanoma, accompanied by strong CD4+ and CD8+ T-cell immunity of magnitudes typically reported for adoptive T-cell therapy [25]. A Phase 2 trial of BNT111 in combination with the anti-PD-1 antibody cemiplimab is ongoing. These divergent outcomes illustrate both the promise and the challenges of off-the-shelf antigen selection, and directly motivated the development of personalized vaccine strategies.

These antigens can cover a certain proportion of the population, enabling the development of “Off-the-Shelf” cancer vaccines. However, due to the strong heterogeneity of tumors and individual variability, the identification of such antigens remains highly challenging and yields only a limited number, making them applicable to only a small population. Consequently, the development of cancer vaccines has gradually shifted toward personalized vaccine strategies.

## 7. Development of Personalized mRNA Cancer Vaccines

In 1998, the pioneer in this field, Steven Rosenberg, published a landmark study in the journal Nature Medicine. His work showed that antigen peptides could be of therapeutic value for people with metastatic melanoma [96]. On the basis of this discovery, Rosenberg’s team examined 26 non-synonymous mutations via whole-exome sequencing (WES) in 2014 [97]. In 2016, his group managed to identify a neoantigen linked to the KRAS (G12D) mutation. After infusion of tumor-infiltrating lymphocytes (TILs) activated by this neoantigen, substantial tumor regression (objective or not) occurred [98].

A new milestone was achieved by the field in 2017. Catherine J. Wu and her group described a personalized vaccine approach in Nature [99]. With RNA sequencing of the genome and of exons plus MHC-I binding predictions as inputs, they located likely neoepitopes of this type. The corresponding neoantigens—long peptides—were made into vaccines; they triggered powerful, long-term responses in the cancer patients studied.

At the AACR Annual 2024, BioNTech published the latest results from its phase I clinical trial (NCT04161755) of Autogene cevumeran, a personalized cancer vaccine for pancreatic ductal adenocarcinoma [16]. This vaccine was developed by BioNTech and is a personalized neoantigen vaccine. It can contain up to 20 neoantigens presented by major histocompatibility complex (MHC) class I and II molecules. The adjuvant is lipid nanoparticles. The phase I clinical trial results showed that among the 16 patients who received the cancer vaccine treatment, eight were identified as positive responders. The study also found that the Autogene cevumeran vaccine can induce long-lasting T-cell expansion. These T cells targeting neoantigens persist in the blood circulation and are expected to have a long-term effect in preventing cancer recurrence.

mRNA-4157 (V940, now designated intismeran autogene) is a personalized mRNA cancer vaccine developed by Moderna in collaboration with Merck. It contains a synthetic mRNA molecule that can encode up to 34 neoantigens [100,101]. In the randomized, open-label, Phase 2b KEYNOTE-942 trial (NCT03897881), 157 patients with completely resected stage IIIB-IV high-risk cutaneous melanoma were randomized 2:1 to receive mRNA-4157 plus pembrolizumab (n = 107) or pembrolizumab monotherapy (n = 50) in the adjuvant setting. In the primary analysis (median [range] 34.9 [25.1–51.0] months), recurrence-free survival (RFS), the primary endpoint, was longer with combination therapy versus monotherapy (hazard ratio [HR] for recurrence or death, 0.510; 95% confidence interval [CI]: 0.288–0.906), with the DMFS (distant metastasis-free survival) hazard ratio was 0.384 (95% CI: 0.172–0.858). The safety profile remained manageable, with no new safety signals [101]. While mRNA-4157 represents the most clinically advanced mRNA cancer vaccine and has received regulatory breakthrough designations, no mRNA cancer vaccine has yet received full regulatory approval, and such approval will depend on confirmatory Phase 3 data.

At the 2026 AACR annual conference, Everest Medicines presented the data from its first human clinical trial (EVM16CX01, NCT06541639). This trial evaluated EVM16 as a single agent or in combination with the PD-1 inhibitor (tislelizumab) for the treatment of advanced solid tumors. The results showed that EVM16 was safe and tolerable in cancer patients, and no dose-limiting toxicities were observed. This clinical trial provided a validation of the clinical application value of EVM16 in immunotherapy by using neoantigens, laying an important foundation for its subsequent clinical development.

With regard to the main pipelines already described, Gritstone Bio has worked on an individualized, heterologous vaccine platform using chimpanzee adenovirus (ChAd68) and self-amplifying mRNA (samRNA) to encode particular neoantigens for each patient. Interim data from the Phase 1 trial (NCT03639714) suggested that the vaccine, used together with anti-PD-1 treatment, has no major toxicities and may provoke T-cell responses specific to neoantigens in patients with advanced solid tumors [30]. In addition, a shared neoantigen vaccine (GRT-C901/CD8-401) that includes 20 neoantigens arising from commonly occurring oncogenic drivers (mutated genes) combined with checkpoint blockade was immunologically effective in Phase 1 trials [29]. Such findings reflect the variety of therapeutic approaches under study clinically.

Even when an mRNA vaccine elicits a measurable T-cell response, clinical benefit is not guaranteed; the response must still operate within a tumor microenvironment (TME) that, in most solid tumors, is actively hostile to effector lymphocytes. The suppressive character of this niche is maintained through multiple, partly redundant mechanisms. Regulatory T cells (Tregs) and myeloid-derived suppressor cells (MDSCs) accumulate within the lesion, tumor-associated macrophages (TAMs) reinforce the local suppressive milieu, and cytokines such as TGF-β and IL-10 act on T cells directly. Collectively, these elements blunt the effector functions of vaccine-induced T cells and favor immune escape, and it is this gap between immunogenicity and clinical efficacy that provides the central rationale for combination regimens.

Besides these pipelines of leading enterprises, mRNA cancer vaccines have entered the clinical trial stage worldwide. As the time for mRNA cancer vaccines approaches, it is expected to make a significant breakthrough in this field and bring new hope to cancer patients.

## 8. Challenges and Prospects

The whole pipeline—beginning with neoantigen identification and continuing through production and clinical use—is fraught with a number of challenges for mRNA cancer vaccines. The present analysis attempts to cover the major obstacles at each stage.

### 8.1. Neoantigen Heterogeneity and HLA Limitations

**Figure 4:**
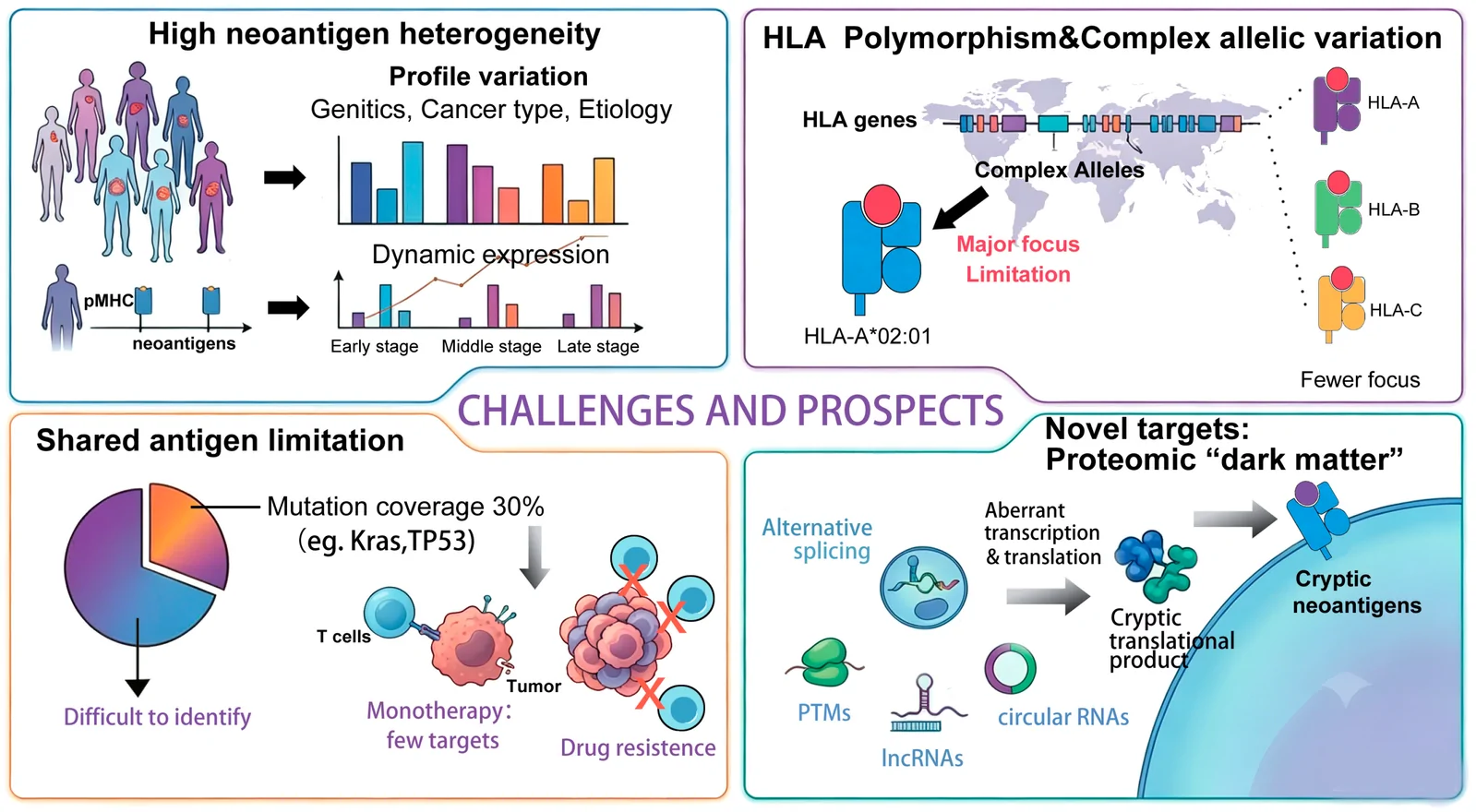
Challenges and prospects of mRNA cancer vaccine. The major challenges are primarily the high heterogeneity of neoantigens and their substantial inter-individual variability. The majority of identified targets are largely confined to the HLA-A*02:01 subtype, whereas other HLA subtypes have received less attention. The coverage rate of shared mutations remains only around 30%. Alternative RNA splicing generates a diverse repertoire of lncRNAs, circRNAs, and various post-translational modifications, all of which contribute to the production of “dark matter”. It is anticipated that more effective and actionable targets may be discovered within these “dark matter” components in the future, offering new avenues for next-generation vaccine strategies. (Figure was drawn in Adobe Photoshop CS5).

Neoantigen repertoires are inherently heterogeneous, reflecting differences in genetic background, cancer type, and underlying etiology [101,102]. This variability is not confined to differences between patients: the pMHC landscape presented by a tumor is highly dynamic and can shift within a single patient as the disease progresses [41] (Figure 4, upper-left). Therefore, antigen selection cannot rely on a static tumor profile. Instead, personalized platforms that tailor vaccines to the individual’s evolving neoantigen expression are needed [42,103], ideally enabling the most suitable antigens to be prioritized in each patient.

The practical bottleneck lies in speed: neoantigens must be identified and characterized, and vaccines against them designed and manufactured, within a clinically meaningful timeframe [43,46]. Overcoming this challenge is a prerequisite for improving clinical efficacy, reducing tumor recurrence, and broadening the application of cancer immunotherapy.

Immunotherapy based on neoantigens represents a highly promising paradigm in the field of oncology. The human leukocyte antigen (HLA/MHC) responsible for peptide presentation exhibits high polymorphism and complex allelic variations. However, most research mainly focuses on the HLA-A*02:01 genotype, which has a phenotypic frequency of approximately 50% in the Caucasian population (Figure 4, upper-right). For instance, the BNT111 vaccine developed by BioNTech combines four shared melanoma-associated tumor antigens (NY-ESO-1, MAGE-A3, tyrosinase, and TPTE) that are restricted to the HLA-A*02:01 allele [44]. Most immunotherapies targeting conventional antigens such as KRAS, MAGE, and NY-ESO-1 are also tailored for HLA-A*02:01 presentation.

These shared antigens (e.g., KRAS and TP53 mutations) cover only 10–30% of the patient population [25,104]. Monotherapies targeting these mutations rarely reach complete eradication and frequently result in drug resistance [105] (Figure 4, lower-left). Furthermore, given the intratumoral heterogeneity and complexity, the probability of different individuals sharing identical neoantigens remains exceedingly low [106]. During the process of tumor occurrence and progression, the interaction between the immune system and tumor cells exerts a strong selective pressure, driving immunoediting and immune escape. This highlights the individualization of MHC molecules restricting immune responses induced by neoantigens. As MHC class I molecules typically bind peptides of 8–11 amino acids in length, identical mutations within the same protein can yield divergent peptide sequences upon processing and presentation across different patients. It is indicated that every individual harbors a unique, specific tumor antigen landscape [107]. Balachandran et al. [33] investigated the 10-year evolutionary trajectories of 70 pancreatic cancer patients and revealed that despite prolonged intervals for mutational accumulation, long-term survivors exhibited recurrent tumors with reduced heterogeneity and a lower burden of mutant neoantigens. This suggests that neoantigen immunogenicity is dictated by two core attributes: non-selfness, determined by sequence similarity to known pathogenic antigens; differential selfness, defined by the structural variance compared to its wild-type counterpart, which is required for T-cell activation. Therefore, precise identification of neoantigens remains the most challenging field in the development of personalized cancer vaccines.

### 8.2. Challenges in mRNA Sequence Design and Optimization

mRNA engineering has advanced considerably, yet several challenges persist in sequence design and manufacturing quality control. Codon optimization remains largely empirical; the interplay between codon usage, mRNA secondary structure, and translation kinetics in primary immune cells is still poorly understood. Capping efficiency at the 5′ end varies between manufacturing batches and methods, and residual uncapped mRNA may trigger innate immune responses that compromise translation. dsRNA byproduct formation during IVT is a persistent quality challenge that demands rigorous purification and control. The optimal combination of nucleotide modifications for cancer vaccines also remains undetermined. Modification levels must balance immunogenicity, stability, and translation, since excessive modification may dampen innate immune stimulation that could benefit anti-tumor immunity. Poly(A) tail length heterogeneity in enzymatically polyadenylated products can likewise lead to variable mRNA stability. Finally, antigen processing signal optimization, such as MHC class I/II trafficking signals and ubiquitination signals, requires further investigation to enhance immunogenicity.

### 8.3. Challenges in In Vivo LNP Delivery

Despite the clinical success of LNP-mRNA vaccines for infectious diseases, delivery challenges specific to cancer vaccines remain: (1) achieving targeted delivery to lymphoid organs (lymph nodes, spleen) rather than the liver remains a challenge; (2) endosomal escape efficiency is low (estimated at 1–2%), representing a major bottleneck for cytoplasmic mRNA delivery; (3) tumor accessibility: the immunosuppressive TME and physical barriers (dense stroma, aberrant vasculature) can limit LNP penetration into tumor tissue; (4) repeat-dose toxicity: chronic administration required for cancer vaccination may lead to accumulation of non-biodegradable lipid components and development of anti-PEG antibodies; (5) manufacturing scalability: while microfluidic mixing enables reproducible LNP production, scale-up to commercial manufacturing while maintaining quality attributes (particle size, PDI, encapsulation efficiency) presents engineering challenges; and (6) stability and storage: LNP-mRNA vaccines require cold chain logistics (−80 °C to −70 °C for current formulations), which limits accessibility in resource-limited settings. Lyophilized formulations and thermostable lipid compositions are under active development.

### 8.4. Dark Matter Neoantigens and Non-Canonical Antigens

Although the application of next-generation sequencing (NGS) enables the precise detection of tumor-specific somatic mutations, this predictive approach fails to accurately identify neoantigens derived from alternative peptide splicing or post-translational modifications (PTMs). The majority of currently identified neoantigens originate from genetic mutations, a subset of which may possess low clonal burden within the tumor, compounding the difficulty of identifying viable targets for cancer vaccines. Despite the tumor-specific advantage of mutation-derived proteins, they do not represent the exclusive source of neoantigens for immunotherapy. Recent evidence demonstrates that aberrant transcription and translation, or cryptic alterations, can generate a vast repertoire of non-canonical proteins that share little homology with normal cellular proteins [108] (Figure 4, lower-right).

In recent years, an increasing number of researchers have turned their attention to this dark matter in proteomics. These studies aim to discover novel and hidden targets for cancer immunotherapy [109]. These cryptic translation products may result from alternative splicing events (including those that generate circular RNAs), translation initiation at non-AUG alternative codons, and ribosomal frameshift errors [110,111]. A large number of studies have shown that during the process of tumor formation, various non-coding RNAs, such as long non-coding RNAs (lncRNAs) and circular RNAs (circRNAs), which were traditionally considered to have no function, can be translated into cryptic proteins in tumors [112]. These proteins are subsequently processed and presented on the cell surface as neoantigens. However, the identification of such neoantigens is still relatively rarely reported. Emerging data suggest that these cryptic proteins may possess superior immunogenicity, making them highly promising candidates for next-generation neoantigens [113].

### 8.5. Manufacturing Scalability and Translational Challenges

Moving personalized mRNA cancer vaccines from clinical trials into routine practice is complicated by obstacles that are as much logistical and regulatory as they are scientific. For a single patient, the complete manufacturing pipeline—from tumor biopsy through sequencing, neoantigen prediction, mRNA design and synthesis, LNP formulation, and quality control to final vaccine release—currently spans 4–8 weeks. Such a schedule is difficult to reconcile with rapidly progressive disease, and the associated logistics of sample transport, sequence-data transfer, and coordination among manufacturing steps add further strain. To shorten this timeline and improve reproducibility, standardized production concepts are now under investigation, including automated in vitro transcription platforms, modular LNP formulation units, and simplified quality testing procedures [114].

Quality assurance poses an additional challenge in individualized production. Every mRNA construct must be verified for sequence accuracy, capping efficiency should reach at least 80%, dsRNA impurities should remain below the limit of detection, and poly(A) tail length together with endotoxin and bioburden data must be documented for each batch. The corresponding LNP parameters—particle size, polydispersity index, zeta potential, and encapsulation efficiency—likewise require characterization, and potency needs to be demonstrated through antigen expression in transfected cells.

Cost and equitable access represent a further translational barrier. Because each vaccine is produced for a single patient, personalized mRNA vaccines will inevitably be more expensive than off-the-shelf therapeutics. Unless manufacturing efficiency is improved, and where possible accompanied by regulatory and health-policy measures, access to these therapies across socioeconomic and geographic groups is unlikely to be equitable [114].

As LNP–mRNA vaccines belong to a broader category of nanomedicine, there is still no jurisdiction that has written dedicated nanomedicine legislation. The United States, the European Union, the United Kingdom, Japan, Australia, Canada, and several Asian countries apply their existing pharmaceutical frameworks instead [115]. Different countries have various approaches. The FDA reviews nano-containing products case by case. The EMA combines nano-specific safety assessment with reflection papers on liposomal, iron-based nano-colloidal and micellar products, and Australia’s TGA treats a nanomedicine simply as a medicine under the Therapeutic Goods Act 1989. None of these countries has created a separate nanomedicine pathway. The European Commission’s 2022 nanomaterial definition (2022/C 229/01) is one of the few cross-cutting reference points, but it is non-binding and still diverges from other legislation. For example, the definition of REACH in the EU emphasizes the particle size distribution, while the US-EPA emphasizes the size range [115]. Given the current situation, it is necessary to establish a regulatory standard that is universal globally.

Encouragingly, nanocarrier-based vaccines have repeatedly navigated these existing pathways successfully. Virosome-based vaccines such as Epaxal (hepatitis A) and Inflexal V (influenza) were registered in Europe as early as the 1990s, demonstrating that rationally engineered nanocarriers—which combine delivery and immunostimulatory functions—can achieve the balance between efficacy and tolerability required for regulatory acceptance [116]. The COVID-19 pandemic subsequently provided a large-scale precedent for LNP-encapsulated mRNA products, with BNT162b2 progressing from emergency use authorization to full approval. Nevertheless, expectations regarding the structure–function characterization of the LNP component, comparability between manufacturing batches, and the control of immunogenicity-related impurities remain areas of active dialog between sponsors and regulators, and the regulatory framework for such products is still evolving rather than simply adapting conventional batch-release rules.

### 8.6. Challenges in Translating Immunogenicity to Clinical Efficacy

Immunogenicity and clinical benefit do not always move in parallel, and reconciling the two remains one of the field’s most persistent difficulties. Clinical trials consistently demonstrate robust neoantigen-specific T-cell responses after mRNA vaccination, yet these responses translate into tumor regression or prolonged survival only inconsistently. Several explanations, by no means mutually exclusive, account for this discrepancy. The antigenic target itself may be unstable: immunoediting can eliminate the very neoantigens against which the vaccine was directed, allowing outgrowth of antigen-negative tumor cells. Even when effector T cells reach the tumor, the local microenvironment often suppresses their function through Tregs, MDSCs, and immunosuppressive cytokines. Tumors may additionally acquire resistance by downregulating MHC expression, impairing the antigen-processing machinery, or engaging alternative survival pathways. Patient selection further complicates interpretation, and criteria based on tumor mutational burden, immune infiltration, and HLA type are still being refined. Treatment setting appears to matter as well: the marked benefit of mRNA-4157 in resected melanoma, relative to the limited efficacy observed in advanced solid tumors, supports the view that adjuvant treatment of minimal residual disease represents a more tractable scenario. Progress will therefore depend on integrated biomarker development, rationally designed combination regimens, and adaptive trial designs capable of accommodating tumor and patient heterogeneity.

## 9. Conclusions

**Figure 5:**
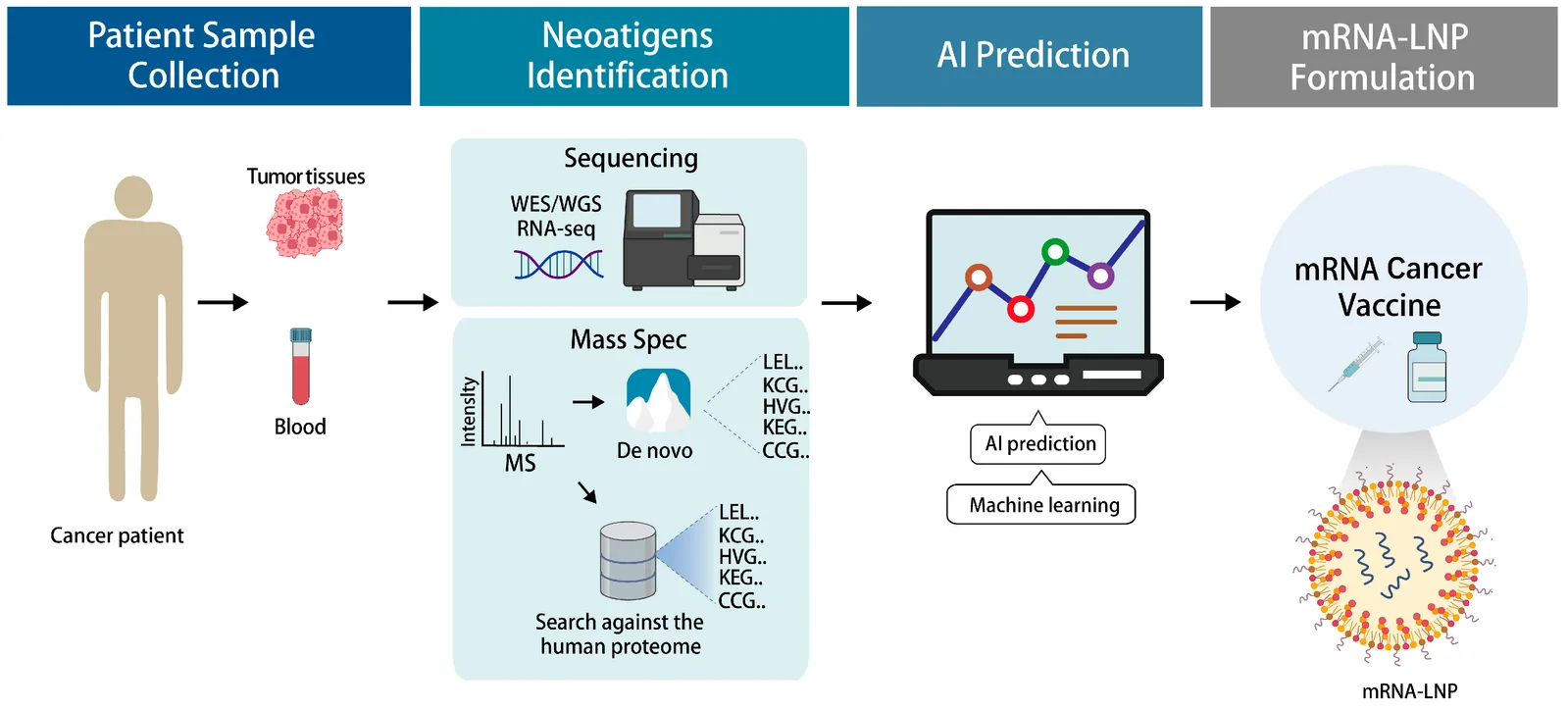
Production process of personalized mRNA cancer vaccine. The process begins with the collection of patient samples. Tumor and normal samples are subjected to whole-exome sequencing (WES)/whole-genome sequencing (WGS)/RNA-seq, and mass spectrometry (MS) to identify somatic mutations and potential neoantigens. Candidate neoantigens are then prioritized using AI-driven algorithms, followed by screening against the human proteome to ensure specificity. Selected neoantigen-encoding mRNA sequences are formulated into lipid nanoparticles (LNPs) to generate the final personalized mRNA cancer vaccine. (Figure was drawn in Adobe Illustrator v29.x).

mRNA cancer vaccines have advanced from a laboratory concept to a clinically testable therapeutic platform, driven by converging progress in neoantigen identification, mRNA engineering, and LNP delivery technology (Figure 5 summarizes the production workflow for personalized mRNA vaccines). Substantial challenges remain nonetheless. Patients differ markedly in neoantigen burden and HLA restriction, and tumors exploit multiple routes of immune evasion. Sequencing- and mass spectrometry-based approaches have sharpened neoantigen prediction, yet both still struggle to capture antigens originating from non-canonical translation events. Encouragingly, successive clinical readouts reported by industry sponsors have continued to demonstrate measurable efficacy of mRNA vaccines in the settings studied. Future progress will hinge on the integration of multi-omics profiling, advanced artificial intelligence algorithms, and optimized LNP formulations; the same developments are expected to extend the reach of mRNA cancer vaccines across a broader range of tumor types.

## Figures and Tables

**Table 1:** Selected mRNA cancer vaccines in clinical trials. (Data verified through ClinicalTrials.gov and primary publications on PubMed, last accessed 8 July 2026. n = number of patients enrolled/vaccinated as reported in the cited primary publications; NR = not reported).

Vaccine (NCT)	Platform	Antigen(s)	Indication	Sponsor	Phase	Combination	n	Primary Endpoint	Status/Key Clinical Outcome
BNT111 (NCT02410733; NCT04526899)	RNA-LPX, i.v. (FixVac)	Shared TAAs (NY-ESO-1, MAGE-A3, tyrosinase, TPTE)	Advanced melanoma	BioNTech	Ph 1 (Lipo-MERIT); Ph 2 ongoing	Anti-PD-1 (cemiplimab) in Ph 2	NR (Ph 1)	Safety; ORR (Ph 2)	Ph 1: durable objective responses in CPI-experienced melanoma; strong CD4+/CD8+ T-cell immunity [25]
BNT122/Autogene cevumeran (NCT04161755)	mRNA-LNP, i.v. (IVAC)	Personalized neoantigens (up to 20; MHC I/II)	Resectable PDAC; solid tumors	BioNTech /Genentech	Ph 1; Ph 2 ongoing	Atezolizumab + mFOLFIRINOX	16 vaccinated (Ph 1 PDAC)	Safety; feasibility	8/16 (50%) T-cell responders; long-lived neoantigen-specific CD8+ T cells persisting years after treatment [26]
mRNA-4157/intismeran autogene (NCT03897881)	mRNA-LNP, i.m.	Personalized neoantigens (up to 34)	Resected high-risk melanoma (adjuvant)	Moderna/ Merck	Ph 2b complete; Ph 3 ongoing	Pembrolizumab (anti-PD-1)	157 (2:1 randomization)	RFS	Primary analysis: RFS HR = 0.561 (95% CI 0.309–1.017; *p* = 0.053), 18-mo RFS 79% vs. 62%; 5-yr descriptive update: RFS HR = 0.510 (0.294–0.887), DMFS HR = 0.411 (0.200–0.843), OS HR = 0.471 [27]
mRNA-5671/V941 (NCT03948763)	mRNA-LNP	Shared neoantigens (KRAS G12D/G12V/G13D/G12C)	KRAS-mutant CRC, pancreatic, NSCLC	Moderna/ Merck	Ph 1	Pembrolizumab	NR	Safety; immunogenicity	Well tolerated; KRAS-specific T-cell responses (interim; full results not published)
CV9202/BI1361849	Protamine-formulated mRNA, i.d. (RNActive)	Shared TAAs (NY-ESO-1, MAGE-C1, MAGE-C2, Survivin, 5T4, MUC-1)	Stage IV NSCLC	CureVac/BI	Ph 1b	Local radiotherapy	26	Safety; tolerability	Well tolerated; antigen-specific immune responses; limited single-agent clinical activity [28]
GRT-C901/GRT-C902 (SLATE)	ChAd68 prime + samRNA boost	Shared neoantigens (20 from driver mutations; KRAS/TP53)	Advanced/metastatic solid tumors	Gritstone bio	Ph 1/2	Ipilimumab + nivolumab	19 (18/19 KRAS-mutant)	Safety; tolerability	Well tolerated; ORR 0%; median PFS 1.9 mo, OS 7.9 mo; TP53-biased T-cell immunodominance observed [29]
Individualized Gritstone vaccine (NCT03639714)	ChAd68 prime + samRNA boost	Personalized neoantigens	Advanced metastatic solid tumors	Gritstone bio	Ph 1	Anti-PD-1	NR (interim analysis)	Safety; tolerability	Well tolerated; neoantigen-specific CD8+ T-cell responses induced [30]
EVM16 (NCT06541639)	mRNA-LNP	Personalized neoantigens	Advanced solid tumors	Everest Medicines	Ph 1	Tislelizumab (anti-PD-1)	NR	Safety; DLTs	Well tolerated; no DLTs; neoantigen-specific T-cell responses observed

**Table 2:** Comparison of peptide, DC, and mRNA cancer vaccine platforms.

Feature	Peptide Vaccines	Dendritic Cell Vaccines	mRNA Vaccines
Manufacturing timeline	2–4 weeks	3–6 months	4–8 weeks
Personalization	Yes (limited epitopes)	Yes (ex vivo loading)	Yes (multiple neoantigens)
Number of epitopes	1–30 peptides	Variable	Up to 34+
Adjuvant required	External adjuvant	Intrinsic (DC maturation)	Intrinsic (LNP adjuvant)
Cost	Low–Moderate	High	Moderate
Storage	Simple (lyophilized, RT)	Complex (cryopreservation)	Cold chain (−80°C)
Repeat dosing	Possible	Limited	Possible
Scalability	Good	Poor	Good (IVT scalable)

## Data Availability

The original contributions presented in this study are included in the article. Further inquiries can be directed to the corresponding authors.
